# An electrochemiluminescence based assay for quantitative detection of endogenous and exogenously applied MeCP2 protein variants

**DOI:** 10.1038/s41598-019-44372-3

**Published:** 2019-05-28

**Authors:** Hannes Steinkellner, Anna Schönegger, Julia Etzler, Prakasha Kempaiah, Anna Huber, Kathrin Hahn, Katrin Rose, Mark Duerr, John Christodoulou, Alexander V. Beribisky, Winfried Neuhaus, Franco Laccone

**Affiliations:** 1 Institute of Medical Genetics, Medical University of Vienna, Waehringer Strasse 10, Vienna, Austria; 2 AIT Austrian Institute of Technology GmbH, Competence Center Health and Bioresources, Molecular Diagnostics, 1210 Vienna, Austria; 3 Department of Medicine, Loyola University Stritch School of Medicine, 2160 South 1st Avenue, Chicago, IL 60153 USA; 4 Meso Scale Discovery, Rockville, Maryland USA; 5 Murdoch Children’s Research Institute, Royal Children’s Hospital, and Department of Paediatrics, University of Melbourne, Melbourne, Australia

**Keywords:** DNA-binding proteins, High-throughput screening

## Abstract

Methyl-CpG-binding protein 2 (MeCP2) is a multifunctional chromosomal protein that plays a key role in the central nervous system. Its levels need to be tightly regulated, as both deficiency and excess of the protein can lead to severe neuronal dysfunction. Loss-of-function mutations affecting MeCP2 are the primary cause of Rett syndrome (RTT), a severe neurological disorder that is thought to result from absence of functional protein in the brain. Several therapeutic strategies for the treatment of RTT are currently being developed. One of them is the use of stable and native TAT-MeCP2 fusion proteins to replenish its levels in neurons after permeation across the blood-brain barrier (BBB). Here we describe the expression and purification of various transactivator of transcription (TAT)-MeCP2 variants and the development of an electrochemiluminescence based assay (ECLIA) that is able to measure endogenous MeCP2 and recombinant TAT-MeCP2 fusion protein levels in a 96-well plate format. The MeCP2 ECLIA produces highly quantitative, accurate and reproducible measurements with low intra- and inter-assay error throughout a wide working range. To underline its broad applicability, this assay was used to analyze brain tissue and study the transport of TAT-MeCP2 variants across an *in vitro* model of the blood-brain barrier.

## Introduction

Human methyl-CpG-binding protein 2 (MeCP2) is a chromosomal protein that acts as a transcriptional repressor and global modulator of gene-expression programs in a DNA-methylation-dependent manner^1^. Although widely expressed throughout different tissue types, MeCP2 plays a specific role in the central nervous system and has been shown to be implicated in various neurological conditions^2^. The protein has two known isoforms, A and B^3^, which are encoded by the *MECP2* gene located on the X-chromosome (Xq28). Loss-of-function mutations of *MECP2* have been found to be the primary cause of Rett syndrome (RTT), a severe neurodevelopmental disorder, which manifests itself during early childhood and affects around 1 in 10,000 live female births^4^. The condition is characterized by a six to 18 months’ period of apparently normal postnatal development followed by a progressive deterioration of acquired cognitive, communication and motor skills, leading to absence of speech, loss of purposeful hand use, replaced by stereotypic hand movements, and gait abnormalities. Additional common signs include the emergence of autistic-like features, abnormal muscle tone, seizures as well as breathing disturbances when the patients are in a waking state^5^.

RTT is rare in males, as *MECP2 de novo* mutations predominantly arise on the paternally derived X-chromosome^6^. Boys carrying mutations that cause classical RTT in females typically present with a distinct neurological condition, namely a severe neonatal encephalopathy, and usually die during their first year of life^7^. Given that the disease-causing gene is subject to X chromosome inactivation (XCI), girls affected with RTT syndrome are somatic mosaics for cells expressing wild-type and mutant MeCP2. The notion that the key features of the disorder can be attributed to insufficient levels of MeCP2 specifically in the central nervous system has recently been corroborated by a mouse model, in which *Mecp2* was silenced throughout peripheral tissues but reactivated at near normal levels within the nervous system, displaying none of the major RTT-like phenotypes^8^. However, not only deficiency but also an excess of the protein causes neuronal dysfunction: while mice overexpressing MeCP2 display seizures and hypoactivity, boys affected with *MECP2* duplication syndrome show severe neurological symptoms that can overlap with those of RTT^9^, highlighting the need for tight regulation of MeCP2 levels in the central nervous system.

There are several indications that RTT syndrome may become a curable condition. Various mouse models have allowed for great insights into RTT pathogenesis, and RTT research has outpaced that of many other neurodevelopmental disorders^1^. Recent findings in the biology of MeCP2 as well as the proof-of-principle demonstration that RTT symptoms are reversible in a Mecp2-deficient mouse model are indeed very promising^10^. Potential therapeutic strategies that are currently being developed include pharmacologic approaches that target signaling pathways downstream of MeCP2, but also approaches that target MeCP2 itself, for instance gene correction, virus-mediated gene delivery, expression of the wild-type *MECP2* allele from the inactive X-chromosome or protein replacement therapy^9^. Given the tight regulation of *MECP2* expression, all of the latter approaches face the challenge of normalizing MeCP2 protein levels within the central nervous system without resulting in a detrimental overdose.

To address this need for highly sensitive and accurate quantification of MeCP2 protein levels, we set out to develop an electrochemiluminescence-based immunoassay (ECLIA) that allows for precise measurement of endogenous as well as exogenous MeCP2 levels in nuclear extracts from different cell lines and mouse tissue samples in a high-throughput format. Furthermore, we used the ECLIA assay described here to test our hypothesis that a recombinant fusion protein consisting of the human MeCP2 isoform B protein and a minimal N-terminal HIV-TAT transduction domain (TAT-MeCP2)^11^ has the potential to cross the blood-brain barrier and to raise the nuclear level of MeCP2 protein in neuronal cells, thus contributing to the further development of a potential protein replacement therapy for RTT.

## Results

### Expression and purification of MeCP2 constructs

**Figure 1:**
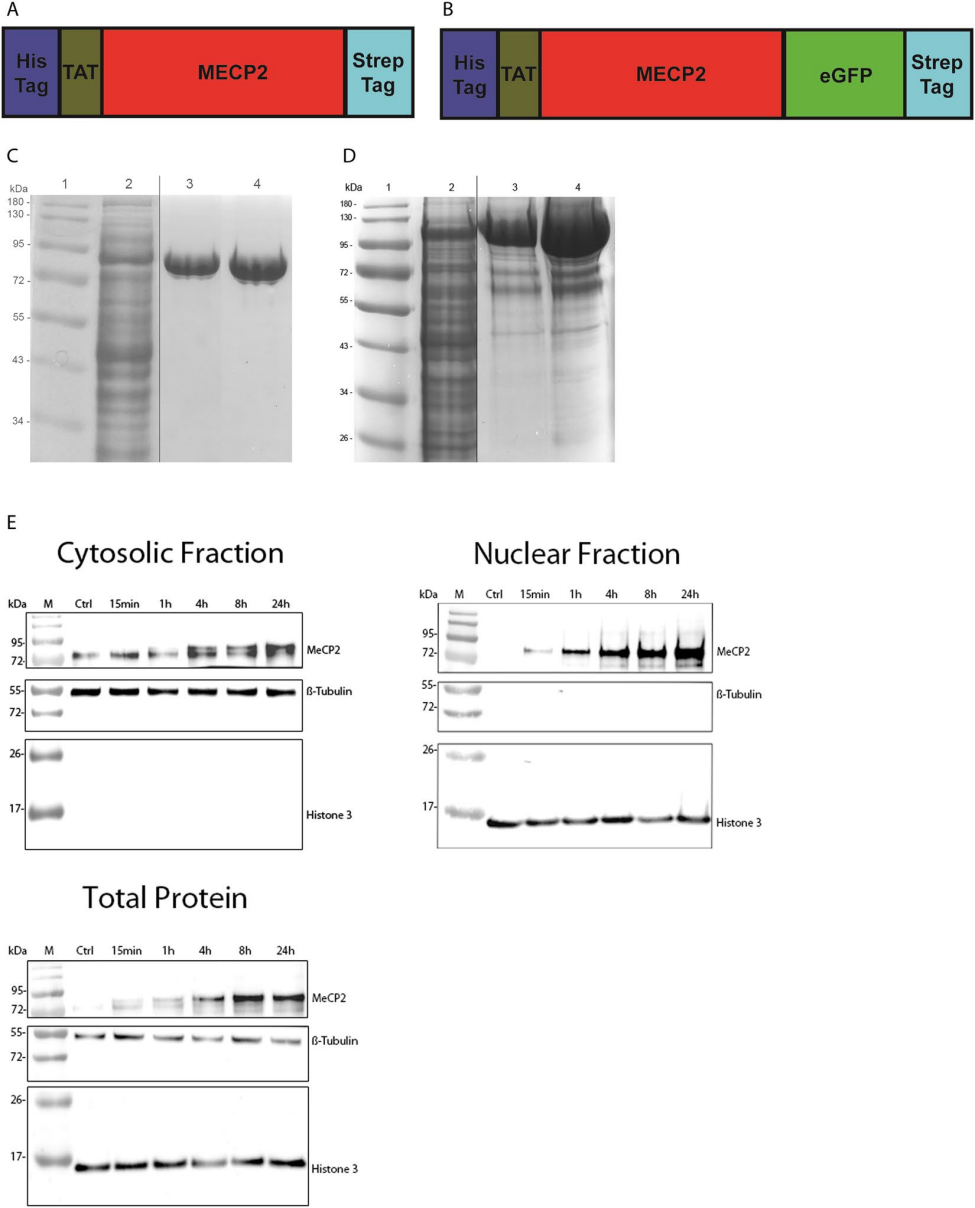
Schematic representation and purification of TAT-MeCP2 fusion proteins. (**A**) TAT-MeCP2 construct map. The first part of this construct codes for the His-tag, followed by the TAT-peptide, MeCP2, and Strep-tag coding regions. (**B**) TAT-MeCP2-eGFP construct map. The first part of this construct codes for the His-tag, followed by the TAT-peptide, MeCP2, eGFP, and Strep-tag coding regions. (**C**) SDS-PAGE of TAT-MeCP2 and (**D**) TAT-MeCP2-eGFP during the various stages of the purification process. Lane 1: Protein size marker. Lane 2: bacterial lysate. Lane 3: TAT-MeCP2 and TAT-MeCP2-eGFP elution fraction following Strep-Tactin XT affinity chromatography. Lane 4: final TAT-fusion protein. The vertical black lines delineate the crop locations. Original, uncropped gels are included in the Supplementary Information (Fig. S4A,B). (**E**) Western blots showing the accumulation of TAT-MECP2 in cytosolic and nuclear fractions as well as total lysate of fibroblasts from a patient carrying the c.806delG mutation over 24 hours. The faint band observed in the cytosolic fraction and total lysate is non-specific, as it was observed in the untreated cells.

The TAT-MeCP2 and TAT-MeCP2-eGFP constructs were designed to encode for the TAT-fusion protein of interest along with sequences encoding for the Strep-tag for Strep-Tactin affinity chromatography (Fig. 1A,B).

Expression of both constructs was carried out in the *E*. *coli* BL21 (DE3) expression strain via induction with IPTG. Protein purification was performed using Strep-Tactin affinity chromatography as the protein capture method, followed by gel filtration chromatography as a polishing step. While both TAT-MeCP2 and TAT-MeCP2-eGFP were already very pure after affinity chromatography, we used gel filtration chromatography to further increase sample purity as well as long-term protein stability.

During SDS-PAGE, both TAT-MeCP2 (60 kDa) and TAT-MeCP2-eGFP (85 kDa) migrate slower than expected for proteins of their size (Fig. 1C,D). This is in line with previous work that shows MeCP2 is an elongated molecule with a higher than expected radius of 6.41 Å^12^. Western blots of cytosolic and nuclear fractions (with β-tubulin and Histone 3 as the two respective house-keeping genes) as well as total lysate show TAT-MeCP2 build-up after 15 minutes in the nuclear fraction and the total lysate, as MeCP2 is predominantly localized to the nucleus. Moreover, TAT-MeCP2 was also found to be present in the cytoplasmic fraction after 4 hours (Fig. 1E).

### Assay specificity, reproducibility and sensitivity

**Figure 2:**
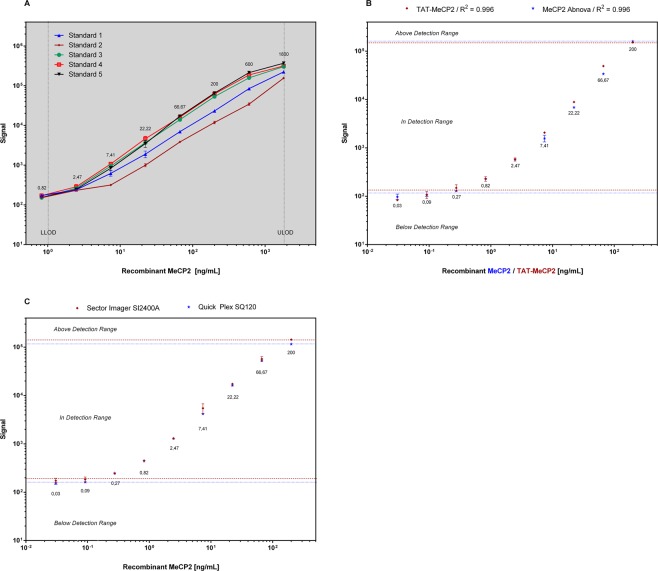
(**A**) Standard curves generated from recombinant human MeCP2 (Abnova) on multiple days. 5-Parameter-Logistic curves in a range from 1.002 to 1800 ng per mL were generated using Discovery Workbench 4.0 software from Meso Scale Discovery (MSD) and GraphPad Prism 6. Error bars show the standard error for each dilution (n = 3). (**B**) Standard curve of recombinant human MeCP2 (blue) and TAT-MeCP2 (red) and (**C**) standard curve of recombinant human MeCP2 generated from two independent sector imagers, SI2400A (blue) and QuickPlex SQ120 (red).

The ECLIA is based on 96-microwell plates with carbon electrodes built in the bottom of the plate for ultra-sensitive detection. The sensitivity of our MeCP2 ECLIA is characterized by a lower limit of detection (LLOD) of 1.002 ng mL^−1^. This value is calculated as 2.5 standard deviations above the blank. Recombinant MeCP2 (Abnova) can be measured over a wide range (1.002 ng mL^−1^ to 1800 ng mL^−1^ with R^2^ = 0.99) with high accuracy and small standard error (Fig. 2A). To further analyze our standard protein (MeCP2, Abnova), we have compared with the standard curves of recombinant TAT-MeCP2 fusion protein (Fig. 2B) and evaluated the standard curve of MeCP2 separately on two different imagers, (sector imager SI2400A and SQ120, MSD) (Fig. 2C). We have also analyzed samples derived from human patient fibroblasts incubated with TAT-MeCP2 for 4, 8 and 24 hours on multiple days showing a CV <9% (data not shown).

The MeCP2 ECLIA described in this paper is able to detect endogenous MeCP2 in a broad range of cell lysates from both mouse and human origin. We measured different cell lysates (human fibroblasts, HEK-293 and NSC-34) on three consecutive days and found an intra-day precision coefficient of variation (CV) ≤6.26% and inter-day precision CV ≤9.71%. Furthermore, we could quantify endogenous MeCP2 levels in brain lysates from wild-type mice. The measurement of mouse brain lysates on three consecutive days yielded an intraday precision CV ≤6.26% (Table 1) and an interday precision CV ≤9.71% (Table 2).

**Table 1:** Intra-assay precision (n = 3 wells per day).

Intra Assay	ng MeCP2/mL protein						
	Well 1	Well 2	Well 3	Mean	SD^a^	SEM^b^	%CV^c^
human fibroblasts	6.42	6.30	6.45	6.39	0.08	0.05	1.24
Hek-293	4.25	4.53	4.82	4.53	0.28	0.16	6.26
Mouse brain lysate	9.92	10.16	10.36	10.14	0.22	0.13	2.17
NSC-34	3.90	3.73	4.05	3.89	0.16	0.09	4.02

^a^SD, standard deviation; ^b^SEM, standard error mean; ^c^CV, coefficient of variation. We used 1–10 µg protein per well of different cell lysates.

**Table 2:** Inter-assay precision (n = 3 separate days) of different isolated lysates from human wild-type fibroblasts, Hek-293 cells, wild-type mouse brain and NSC-34 cells.

Inter Assay	ng MeCP2/mL protein			
	Mean	SD^a^	SEM^b^	%CV^c^
human fibroblasts	6.61	0.64	0.37	9.71
Hek-293	4.18	0.31	0.18	7.39
Mouse brain lysate	11.22	0.94	0.54	8.33
NSC-34	3.82	0.33	0.19	8.75

^a^SD, standard deviation; ^b^SEM, standard error mean; ^c^CV, coefficient of variation. We used 1–10 µg protein per well of different cell lysates.

### Evaluation of MeCP2 protein levels in mouse brains and human fibroblasts

**Figure 3:**
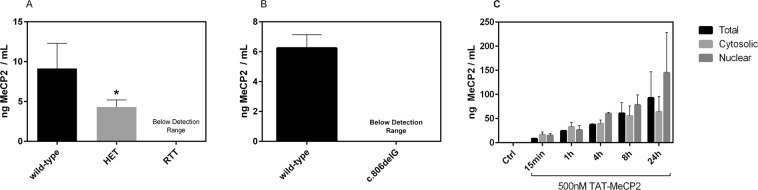
Determination of Mecp2 protein levels in mouse brains and human fibroblasts via MeCP2 ECLIA assay. (**A**) Total Mecp2 protein levels in brain nuclear lysates from female wild-type and heterozygous mice (n = 4) and one RTT mouse. The data presented are mean ± SD of triplicate wells (n = 3). HET, heterozygote; RTT, Mecp2-knock out mouse; (**B**) MeCP2 protein levels were measured in cell lysates from fibroblast cell lines isolated from a patient carrying the c.806delG mutation and healthy control. The data presented are mean ± SD of triplicate wells (n = 3). (**C**) Build-up of TAT-MECP2 levels in the cytosolic (light grey) and nuclear (dark grey) fractions as well as total (black) lysate in c.806delG cells over 24 hours as measured by ECLIA. The TAT-MECP2 concentration used in this experiment was 500 nM. The cells were then washed with DPBS and treated with 0.05% trypsin-EDTA for five minutes to remove extracellular bound TAT-MeCP2 fusion protein. Media with serum was then added to inactivate trypsin and the pellet was collected by centrifugation at 500 *g* for five minutes. The pellet was washed twice with ice-cold DPBS and sample preparation was carried out as described in the methods section.

We observed reduced Mecp2 protein levels in brain lysates of a heterozygote (*Mecp2*^+/−^) compared to wild-type mouse. As expected, we could not detect any Mecp2 protein in brains of knock-out mice (*Mecp2*^−/y^) with our assay (Fig. 3A). Furthermore, we assessed the MeCP2 protein levels in cell lysates from human wild-type and MeCP2-deficient fibroblast cell lines and found MeCP2 levels in the latter cell line (c.806delG) to be below the detection range (Fig. 3B).

### Time-dependent uptake of TAT-MeCP2 fusion protein

To demonstrate that our MeCP2 ECLIA is suitable to measure recombinant human TAT-MeCP2 fusion protein *in vitro*, we incubated a human c.806delG mutant fibroblast cell line with 500 nM recombinant TAT-MeCP2 and then collected cells at stipulated time points for analysis. We observed a time-dependent increase in protein levels over 24 hours with the highest TAT-MecP2 amounts accumulating in the nuclear fraction (Fig. 3C).

### Transport of TAT-MeCP2 and TAT-MeCP2-eGFP across a BBB *in vitro* model

**Figure 4:**
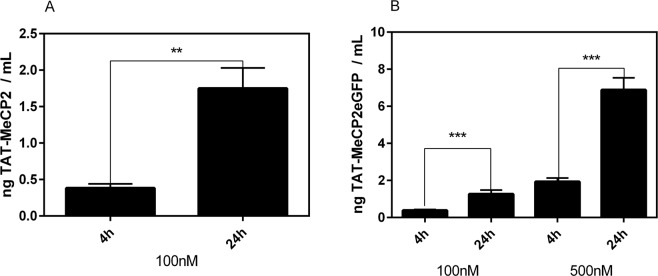
Transport of TAT-MeCP2 and TAT-MeCP2-eGFP across a blood-brain barrier *in vitro* model. Mouse brain endothelial cerebEND cells were cultured on a semipermeable membrane in a transwell model and the transport of (**A**) 100 nM TAT-MeCP2 (n = 3) and (**B**) 100 or 500 nM TAT-MeCP2-eGFP (n = 4) from the apical to the basolateral chamber was studied. Samples were taken after four and 24 h, respectively, and protein levels were quantified by the MeCP2 ECLIA assay. Validation of the MeCP2 ECLIA assay.

We confirmed that the MeCP2 ECLIA is capable of measuring different TAT-MeCP2 batches in a concentration-dependent manner. As a further application of the assay, we used an established *in vitro* BBB model to study the transport of TAT-MeCP2 and TAT-MeCP2eGFP fusion proteins across mouse brain endothelial cell layers. Our results showed that both TAT-MeCP2 (Fig. 4A) and TAT-MeCP2eGFP (Fig. 4B) can be readily detected in the basolateral compartment after 4 h (0.382 ng mL^−1^ TAT-MeCP2 of 100 nM apically applied TAT-MeCP2; 0.382 ng mL^−1^ TAT-MeCP2-eGFP of 100 nM apically applied TAT-MeCP2-eGFP). TEER values before and after the transport studies confirmed tight cell layers throughout the experiments.

## Discussion

Here we report on the expression and purification of TAT-MeCP2 fusion proteins and the development of a 96-well plate electrochemiluminescence immunoassay (ECLIA) to measure endogenous MeCP2 and recombinant TAT-MeCP2 fusion protein levels. MeCP2 is an abundant chromosomal protein and is one of the members of the methyl-CpG-binding domain protein (MBD) family, which is functionally involved in chromatin remodeling and transcriptional regulation. There are two crucial domains in MeCP2. One is the MBD and another is the transcriptional repression domain (TRD), which can recruit different protein partners, such as HDACs and Sin3A, to form a transcriptional repression complex and regulate target gene expression^13,14^. Loss of MeCP2 protein function has been shown to cause RTT syndrome, treatment for which is currently limited to physical therapy and symptom management. Importantly, the identification of a common genetic cause in most cases, a relatively well-defined history, and extensive neurobiological data from postmortem and animal studies make RTT a good candidate for the development of targeted treatments^15,16^.

One potential strategy to titrating MeCP2 up to its appropriate concentration is protein replacement therapy. Over the last two decades it has been shown that TAT fusion proteins are capable of crossing the BBB and that they might have the potential to be used for the treatment of neuronal diseases^17–20^. Here we demonstrate for the first time, the expression and purification of milligram amounts of pure and stable TAT-MeCP2 fusion protein variants (Fig. 1). The challenges associated with this work include ensuring homogeneous and ongoing delivery of the appropriate levels across the BBB, as well as an adequate cell penetration and localization of the supplied MeCP2 to the nucleus^9^.

To assess the potential of TAT-fusion proteins and other therapeutic approaches that aim to restore normal MeCP2 protein levels, there is a strong need for an assay that can measure MeCP2 levels in a time-efficient, low-cost and high-throughput manner. As such, our MeCP2 ECLIA is a novel, highly sensitive assay, capable of measuring MeCP2 amounts in a highly quantitative, accurate and reproducible manner, with low intra- and inter-assay variability in human and mouse samples (Tables 1 and 2).

Until recently, semi-quantitative western blot and commercially available MeCP2-ELISA’s were the only available methods to assess MeCP2 protein levels. Compared to western blot, our MeCP2 ECLIA is less time consuming as well as less laborious. We quantified MeCP2 protein levels of various wild type, heterozygous and MeCP2 knock out mouse brain samples by the ECLIA (Fig. 3A) as well as by western blot (Fig. S3) and observed generally similar results. However, there was a significant difference in MeCP2 levels of female wild-type and heterozygous mouse brain samples measured by our ECLIA that was not detected as statistically significant by western blot. We propose that the improved accuracy of the MeCP2 ECLIA might play an important role in the search for novel compounds that can increase MeCP2 protein levels. Furthermore, our MeCP2 ECLIA is cheaper than commercially available MeCP2-ELISA’s and showed a broad dynamic range from 1.002 ng mL^−1^ to 1800 ng mL^−1^ with R^2^ = 0.996, allowing for even small-scale experimental samples to be quantified. Of note, the detection limit of both methods is based on the anchoring of calibrators, affinities of antibodies, and various detection methods lead to different absolute quantitation and calculated limits of detection.

Finally, we compared our novel ECLIA to all the commercially available ELISA kits and found that our ECLIA greatly outperformed the latter. Our MeCP2 ECLIA showed a broad dynamic range from 1.002 ng mL^−1^ to 1800 ng mL^−1^, whereas the stated working range of the ELISA kits was smaller from 0.312 ng mL^−1^ to 20 ng mL^−1^ (mouse, Cloud-Clone Corp.) and 0.156 ng mL^−1^ to 10 ng mL^−1^ (human, Cloud-Clone Corp.). Moreover, the ECLIA is able to measure samples from mouse and human origin in the same assay format. In line with other findings, our assay also detected highest MeCP2 levels in mouse brain lysates followed by NSC-34 and human hepatocellular carcinoma cell line (HePG2) (data not shown). The lack of consistent definition of the standard of limit of detection precludes a direct lower limit comparison between the ECLIA and ELISA.

*MECP2* mutations are complex and heterogeneous and vary widely including point mutations, duplications, insertions, small and large, or whole *MECP2* gene deletions^21^. In order to investigate MeCP2 protein levels in the affected individuals, we chose a MeCP2-deficient fibroblast cell line (c.806delG) derived from a male patient with neonatal encephalopathy acting as a model for RTT syndrome. MeCP2 protein was not detected in this cell line by immunofluorescence (Fig. S1) and was also found to be below the detection limit in our assay (Fig. 3A,B) showing the accuracy of our ECLIA system and demonstrating its efficiency for uptake studies with TAT-MeCP2 fusion protein. The incubation of a MeCP2-deficient cell line (c.806delG) with TAT-MeCP2 resulted in a concentration dependent increase of MeCP2 protein levels, indicating the potential of TAT-MeCP2 fusion protein to replace endogenous MeCP2 in RTT patients. Finding ways to increase MeCP2 protein levels in RTT patients is one of the main strategies that could overcome RTT syndrome. However, for all of those therapeutic approaches, protein dosage is an important concern, as it has been shown that excessive levels of MeCP2 may cause neurological symptoms seen in patients suffering from Xq28 duplication syndrome and in mice overexpressing the protein^22–24^. Thus, our MeCP2 ECLIA represents a useful tool for determining the optimum quantity of exogenously applied MeCP2 fusion proteins.

## Conclusion

In this work, we report for the first time on successful expression and purification of recombinant TAT-MeCP2 fusion proteins. In addition, we developed a new ECLIA, representing a quantitative tool to investigate appropriate dosage of MeCP2 in neurons of the murine brain and to monitor the protein behavior in these cells. Furthermore, we confirmed the applicability of the assay for permeation studies of recombinant TAT-MeCP2 fusion proteins across a mouse BBB *in vitro* model.

## Materials and Methods

### Antibodies for MeCP2 ECLIA

The primary mouse monoclonal capture antibody directed against the C-terminus (amino acids 471–486) of human MeCP2 was purchased from Sigma-Aldrich (clone Mec-168, #M6818, St. Louis, MO); the secondary anti-MeCP2 rabbit polyclonal capture antibody was custom-made by Eurogentec (Seraing, Belgium); The third antibody which was used as the detection antibody was goat anti-rabbit HRP-Sulfo TAG from Meso Scale Discovery (MSD, Gaithersburg, US). Recombinant human full length MeCP2 standard protein (0.06 mg mL^−1^) was purchased from Abnova (#H00004204-P01, Taipei City, Taiwan).

### Expression and purification of TAT-MeCP2 and TAT-MeCP2eGFP fusion protein

The TAT-MeCP2 construct consists of the sequences encoding from 5′ to 3′, the His-tags, TAT-peptide, human MeCP2 isoform B (498aa), and Strep-tag cloned into the expression plasmid pET-28a(+) (Novagen, Merck, Darmstadt, Germany). Protein expression was carried out in the *Escherichia Coli* strain BL21 (DE3) (#60300-2, Lucigen) in eight 300 mL LB Rich cultures containing 50 mg mL^−1^ Kanamycin (#25389-94-0, Roth). Following induction with 0.5 mM isopropyl-b-D-1-thiogalactopyranoside (IPTG, #I6758, Sigma Aldrich), these cultures were incubated for 20 hours at 30 °C shaking at 250 RPM. Cells were harvested by centrifugation at 4200 *g* for 30 min; the pellets were stored at −80 °C.

The protein was purified in a step-wise process involving cell disruption via sonication, centrifugation to pellet cell debris, Strep-Tactin XT affinity chromatography (#2-4014-001, IBA, Göttingen), gel filtration chromatography and LPS removal using Triton X-114 (X114, Sigma-Aldrich). The purified TAT-MeCP2 protein samples were stored at −80 °C.

The TAT-MeCP2-eGFP construct was designed, expressed and purified in a similar fashion to its TAT-MeCP2 counterpart, with the only difference being the presence of eGFP coding sequence between the sequences encoding for MECP2 and the Strep-Tag. The coding sequences of both constructs can be provided upon request.

### Cell culture and sample preparation

A Human dermal fibroblast (HDF) cell line from a male, healthy control donor (#AG21708) was obtained from Coriell Cell Repositories (CCR, Coriell Institute for Medical Research, NJ, USA). HDF cells from a male patient with neonatal encephalopathy carrying a truncation mutation (c.806delG) were established^25^.

Approval for skin biopsy procurement for research purposes was obtained from the Human Research Ethics Committee of the Children’s Hospital at Westmead, Australia. HDF cell lines and Hek-293 cells were cultured in Dulbecco’s modified Eagle’s medium (DMEM, #41966 from Fisher Scientific) supplemented with 10% foetal bovine serum (FBS, #F9665 Sigma-Aldrich, Germany) and 1% penicillin/streptomycin (Gibco/Life Technologies, Darmstadt, Germany).

The mouse motor neuron-like hybrid cell line (NSC-34, #CLU140-A, Tebu-Bio) was cultured in DMEM without sodium pyruvate (#41965 from Fisher Scientific) supplemented with 10% FBS and 1% penicillin/streptomycin. Mouse brain endothelial cell line cerebEND was cultured in DMEM supplemented with 10% FBS and 1% penicillin/streptomycin in 0.5% gelatine coated cell culture tissue flasks.

For cytosolic and nuclear protein extraction, cells were harvested and washed twice with DPBS, centrifuged at 500 *g* for 5 min and the protein was extracted from adherent cells as described in Suzuki *et al*.^26^ with slight modifications.

For uptake studies with TAT-MeCP2 fusion protein, HDF cells were plated on 6-well plates or 100 mm dishes (Greiner-Bio One, Kremsmünster, Austria) overnight before adding different concentrations of TAT-MeCP2 and were incubated for various time points. The cells were then treated with 0.05% trypsin-EDTA solution (Fisher Scientific) for five min and washed with DPBS twice. For sample preparation, the cell pellets were dissolved in ice-cold lysis reagent (1X tris-buffered saline pH 7.4, 1% Triton X-100, 1 mM ethylenediaminetetraacetic acid, 1 mM ethylene glycol tetraacetic acid, 0.05% sodium dodecyl sulfate, 0.1% sodium deoxycholate, and 2% nonidet-P40) with freshly added protease inhibitors (1 mM phenylmethylsulfonyl fluoride [from Sigma], 5 mM sodium fluoride [Merck Chemicals], 1 mM sodium orthovanadate, protease inhibitor cocktail [#PI8340 from Sigma Aldrich]) and incubated on ice for about 25 min before centrifugation at 10000 *g* for 12 min at 4 °C. The supernatant was analyzed by the BCA Protein Assay Kit (Thermo Scientific Pierce) to quantify the protein concentration.

### Animal experiments

The B6.129P2(C)-Mecp2^tm1.1Bird^/J mouse strain (Jackson Laboratory), described in^27^, was housed in the animal facility at the Department of Biomedical Research, Medical University of Vienna, Austria. *Mecp2* mutant hemizygous males (*Mecp2* -/Y) and wild type littermates were obtained by mating heterozygous females with wild-type males (C57BL/6 J). All mice were bred under the same conditions with 12 h light cycle and provided with water and food *ad libitum*. The ear was tagged for genotyping. Consent was obtained from the Bundesministerium für Wissenschaft und Forschung und Wirtschaft (the Austrian Federal Ministry of Science, Research and Economy) for the animal experiments, which were performed in accordance with local animal welfare regulations (GZ: 66.009/0218-II/3b/2015).

### Genotyping

Genomic DNA was extracted from mouse ear punches using Qiagen’s DNeasy Blood & Tissue DNA extraction kit (Qiagen, #69506) according to the manufacturer’s instructions. The DNA was analyzed by hot-start polymerase chain reaction (PCR) using primers as follows: 5′-AAATTGGGTTACACCGCTGA-3′, 5′-CCACCTAGCCTGCCTGTACT-3′ and 5′-CTGTATCCTTGGGTCAAGCTG-3′. Genotyped samples were loaded on Midori Green Advance (#617004, Biozym, Austria) agarose gels (2%) and imaged under UV light (Fig. S2).

### Sample preparation of mouse brain lysates

The mice were sacrificed and following excision, whole brains were washed twice in ice-cold phosphate-buffered saline (PBS, pH 7.4), immediately frozen in liquid nitrogen, placed on dry ice and stored at −80 °C until lysate preparation.

Total mouse brains were weighed and immediately dissolved in 1 mL of ice cold hypotonic lysis reagent (10 mM HEPES, pH 7.9, with 1.5 mM magnesium chloride (MgCl_2_), 10 mM potassium chloride (KCl) with freshly added 1 mM 1,4-dithiothreitol (DTT, #111474, Merck-Millipore) and protease inhibitor cocktail (#PI8340 from Sigma Aldrich)) per 100 mg tissue and homogenized with a glass tissue homogenizer (15 strokes A and 15 strokes B). Following incubation on ice, the cell lysates were centrifuged at 10000 *g* for 20 min at 4 °C. The supernatant was transferred to a new tube (cytoplasmic fraction) and the nuclei pellet was re-suspended in 140 µL extraction buffer (20 mM HEPES, pH 7.9, with 1.5 mM MgCl_2_, 0.42 M NaCl, 0.2 mM EDTA, 25% (v/v) Glycerol with freshly added 1 mM 1,4-dithiothreitol (DTT, #111474, Merck-Millipore) with protease inhibitor cocktail (#PI8340 from Sigma Aldrich)) per 100 mg of starting tissue and was shaken gently for 30 min on a precooled block. The extracted nuclei were then centrifuged at 16000 *g* for 10 min at 4 °C. The supernatants were transferred to a new pre-chilled tube (nuclear fraction) and protein concentration was determined by using standard procedures using Bradford-Reagent (BioRad Reagent, BioRad, Austria).

### MeCP2 ECLIA protocol

96-Well Multi-Array High Bind plates (#L15XB, Meso Scale Discovery, MSD) were coated with monoclonal mouse anti-MeCP2 antibody. The plates were incubated overnight at 4 °C. On the next day, all necessary reagents were warmed to 25 °C and the plates were blocked with 125 μL of blocking solution (MSD Blocker A) for 90 min at room temperature and subsequently washed three times with PBS–Tween (0.05%). Afterwards, samples (25 μL per well diluted in 1% MSD Blocker A or cell lysis buffer) were added and the plates were incubated at room temperature for another 90 min. Polyclonal rabbit anti-MeCP2 antibody (1:6000 in 1% MSD Blocker A) was added to each well for 4 h followed by 0.75 μg mL^−1^ of MSD Sulfo-TAG goat anti-rabbit detection antibody in 1% MSD Blocker A. After a one-hour incubation at room temperature with gentle agitation, free detection antibody was removed from the plate by washing three times. Finally, 150 μL Tris-based Read Buffer T (1X) with surfactant (MSD) containing tripropylamine as a co-reactant for light generation was added to the plate. Upon electrochemical stimulation, the ruthenium label bound to the carbon electrode emitted luminescence light at 620 nm. ECL signals were captured by a built-in charge-coupled device (CCD) camera in a Sector Imager 2400 reader (MSD) and recorded as signal counts.

### Permeation studies of recombinant TAT-MeCP2 fusion proteins

To study the transport of recombinant TAT-MeCP2 and TAT-MeCP2-eGFP proteins across the blood-brain barrier (BBB) *in vitro*, the established mouse BBB model based on cerebEND cells was utilized. These cells were a kind gift from Prof. Carola Förster and were described for the first time in 2006^28^. CerebENDs were seeded with a density of 40000 cells/cm^2^ on collagen IV coated 12-well inserts and were cultured in Dulbecco’s Modified Eagle’s Medium (DMEM) supplemented with 10% FCS and 1% penicillin/streptomycin as recently described^29^. The medium was exchanged every other day, and 100 nM hydrocortisone was also supplied on days 7 to 13. On day 13, 100 or 500 nM of TAT-MeCP2 or TAT-MeCP2-eGFP were applied in the apical compartment and inserts were transferred in new, pre-warmed 12-wells filled with growth medium after 4 h. Samples were taken after 4 h and 24 h and analyzed by the novel MeCP2 ECLIA. In addition, transendothelial electrical resistance before and after the experiments was measured in order to ensure cell layer integrity.

### Statistical analysis

Statistical analysis of the quantitative experimental data was performed with GraphPad Prism 6 software. Results are presented as means ± SD or means ± SEM. Statistical significance was examined using the Mann-Whitney U test. Significant differences are marked in the figures with *(p < 0.05), **(p < 0.01) and ***(p < 0.001).

## Supplementary information

Supplementary Info

## Data Availability

All data will be provided upon request.
