# Supplementary material for: An electrochemiluminescence based assay for quantitative detection of endogenous and exogenously applied MeCP2 protein variants
Source: Sci Rep. 2019 May 28;9:7929. doi: 10.1038/s41598-019-44372-3 (PMC6538716; doi:10.1038/s41598-019-44372-3)
Supplement: Supplementary file 1 — Supplementary Info [file 41598_2019_44372_MOESM1_ESM.pdf]

Supporting Information (Fig. S1–S4) of Steinkellner et al. "An electrochemiluminescence based assay for quantitative detection of endogenous and exogenously applied MeCP2 protein variants"

Hannes Steinkellner[1*], Anna Schönegger[1], Julia Etzler[1], Prakasha Kempaiah[3], Anna Huber[1], Kathrin Hahn[2], Katrin Rose[1], Mark Duerr[4], John Christodoulou[5], Alexander V. Beribisky[1], Winfried Neuhaus[2], Franco Laccone[1]

A

B

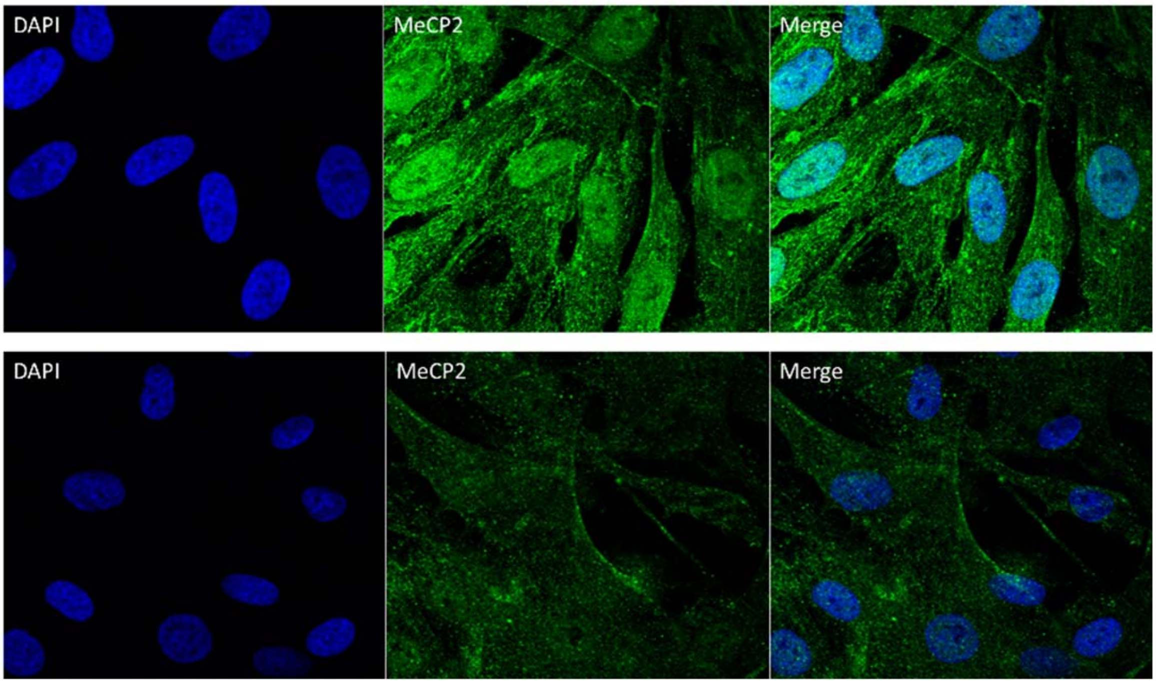

Fig. S1 A/B. Immunofluorescent images of cultured fibroblasts to compare MeCP2 levels in healthy control fibroblasts **(A)** and a RTT patient fibroblast (c.806delG) cell line **(B)**. Immunofluorescence was performed using the anti-MeCP2 rabbit polyclonal antibody that was used as capture antibody in our MeCP2-ECLIA (Eurogentec, Seraing, Belgium). Nuclei were counterstained with DAPI.

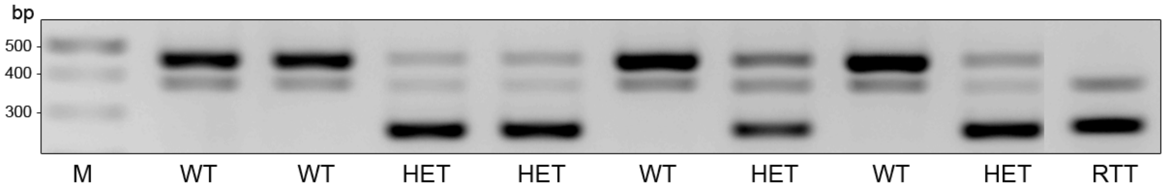

Fig. S2. Agarose gel of genotyping samples from female wild type and heterozygous mice and one male knock out mouse. M (Marker), WT (female wild type mouse, *Mecp2*$^{+/+}$), HET (female heterozygous mouse, *Mecp2*$^{+/-}$) and RTT (Mecp2 null male, *Mecp2*$^{-/y}$). Expected results as specified by the supplier: Mutant = 240 bp; Heterozygote = 240 bp and 465 bp; Wild type = 465 bp

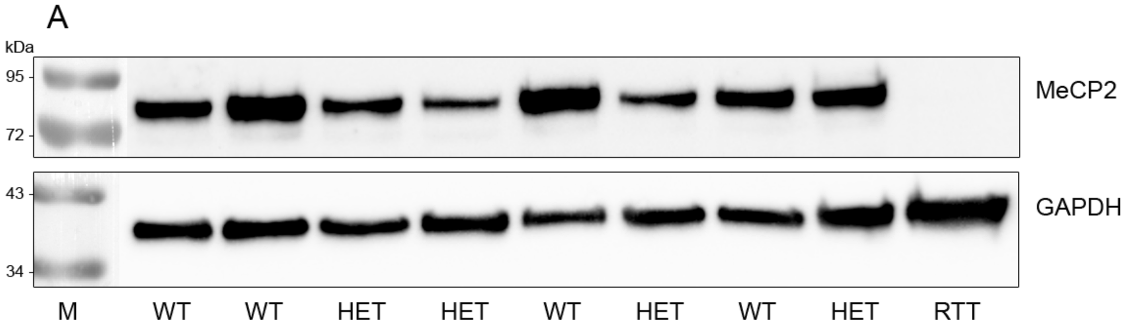

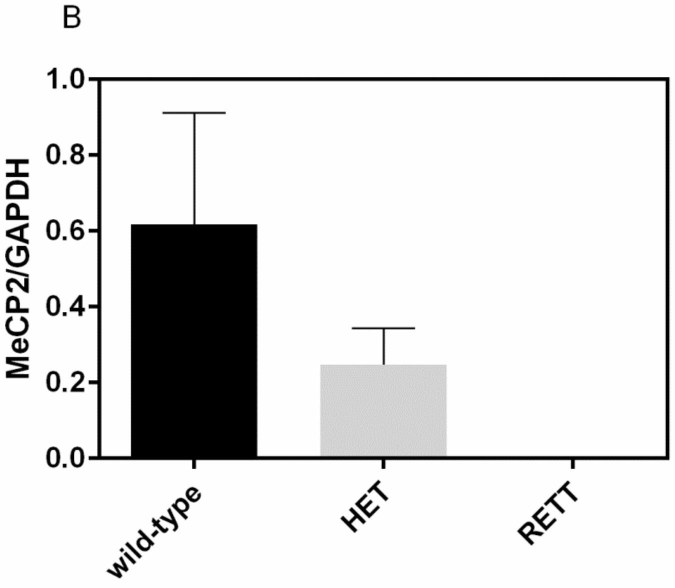

Fig. S3 A/B. Western blot analysis of wild-type (WT), heterozygous (HET) and Mecp2-Knock out (RTT) mice. **(A)** Immunoblotting was performed using the anti-MeCP2 rabbit polyclonal antibody that was used as capture antibody in our MeCP2-ECLIA (Eurogentec, Seraing, Belgium). **(B)** Quantification of MeCP2 western blot normalized to GAPDH. No significant difference between female wild-type and heterozygous mice was detected.

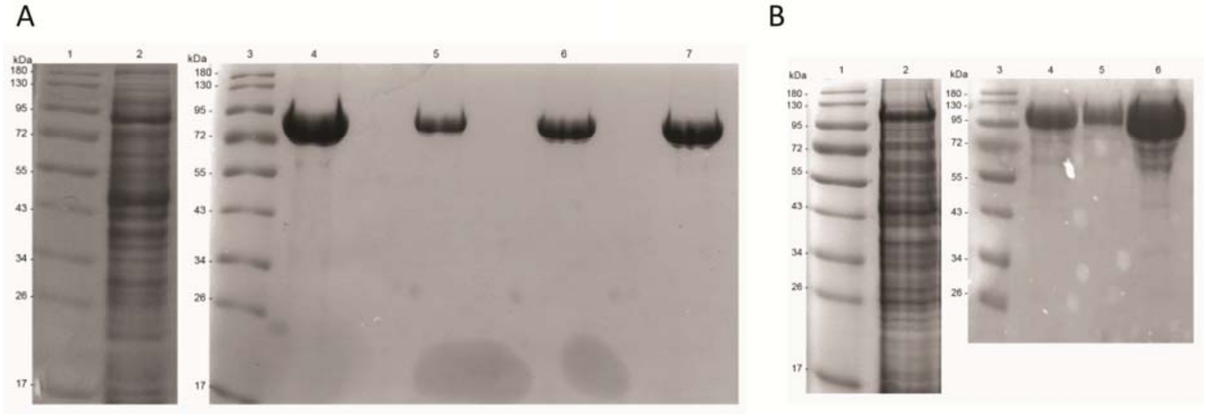

Fig. S4 Uncropped SDS-PAGE gels. TAT-MECP2 **(A)** and TAT-MECP2-eGFP **(B)**
